# Lymphatic Vessels and Their Surroundings: How Local Physical Factors Affect Lymph Flow

**DOI:** 10.3390/biology9120463

**Published:** 2020-12-11

**Authors:** Eleonora Solari, Cristiana Marcozzi, Daniela Negrini, Andrea Moriondo

**Affiliations:** Department of Medicine and Surgery, University of Insubria, I-21100 Varese, Italy; eleonora.solari@uninsubria.it (E.S.); cristiana.marcozzi@uninsubria.it (C.M.); daniela.negrini@uninsubria.it (D.N.)

**Keywords:** lymphatic vessels, intrinsic contractility, extrinsic mechanism, hydraulic pressure, shear stress, lymph flow, osmolarity, tissue temperature

## Abstract

**Simple Summary:**

Lymphatic vessels are responsible for the drainage of liquids, solutes, and cells from interstitial spaces and serosal cavities. Their task is fundamental in order to avoid fluid accumulation leading to tissue swelling and edema. The lymphatic system does not possess a central pump, instead lymph is propelled against an overall hydraulic pressure gradient from interstitial spaces to central veins thanks to two pumping mechanisms, which rely on extrinsic forces or the intrinsic rhythmic contractility of lymphatic muscle cells embedded in vessel walls. This latter mechanism can very rapidly adapt to subtle changes in the microenvironment due to hydraulic pressure, lymph flow-induced wall shear stress, liquid osmolarity, and local tissue temperature. Thus, endothelial and lymphatic muscle cells possess mechanosensors that sense these stimuli and promote a change in contraction frequency and amplitude to modulate lymph flow accordingly. In this review, we will focus on the known physical parameters that can modulate lymph flow and on their putative cellular and molecular mechanisms of transduction.

**Abstract:**

Lymphatic vessels drain and propel lymph by exploiting external forces that surrounding tissues exert upon vessel walls (extrinsic mechanism) and by using active, rhythmic contractions of lymphatic muscle cells embedded in the vessel wall of collecting lymphatics (intrinsic mechanism). The latter mechanism is the major source of the hydraulic pressure gradient where scant extrinsic forces are generated in the microenvironment surrounding lymphatic vessels. It is mainly involved in generating pressure gradients between the interstitial spaces and the vessel lumen and between adjacent lymphatic vessels segments. Intrinsic pumping can very rapidly adapt to ambient physical stimuli such as hydraulic pressure, lymph flow-derived shear stress, fluid osmolarity, and temperature. This adaptation induces a variable lymph flow, which can precisely follow the local tissue state in terms of fluid and solutes removal. Several cellular systems are known to be sensitive to osmolarity, temperature, stretch, and shear stress, and some of them have been found either in lymphatic endothelial cells or lymphatic muscle. In this review, we will focus on how known physical stimuli affect intrinsic contractility and thus lymph flow and describe the most likely cellular mechanisms that mediate this phenomenon.

## 1. Introduction

**Figure 1:**
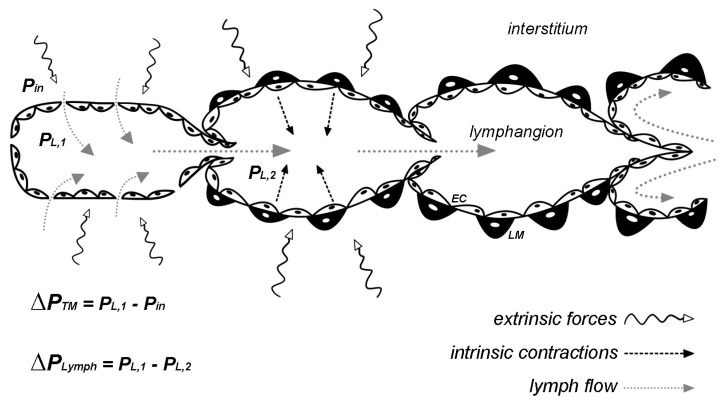
Functional scheme of how lymph is drained and propelled. Extrinsic forces and/or intrinsic rhythmic contractions generate transmural pressure gradients (**Δ*P_TM_***) across the wall of initial lymphatic capillaries and intraluminal pressure gradients (**Δ*P_Lymph_***) between adjacent lymphangions. Intraluminal valve leaflets are devoid of LM. (***P_in_***) interstitial hydraulic pressure; (***P_L_***) intraluminal hydraulic pressure; (***EC***) lymphatic endothelial cells; (***LM***) lymphatic muscle cells.

The lymphatic system drains fluid, macromolecules, and cells from the surrounding interstitium and from serosal cavities, thus contributing to tissue homeostasis and fluid balance [1,2]. Lymph enters blind-ended lymphatic capillaries by means of overlapping endothelial cells forming primary unidirectional valves [3,4,5] and then it is propelled afferently through collecting vessels, lined with muscle cells in their walls and with intraluminal unidirectional valves [6] delimiting adjacent lymphangions, the lymphatic functional units (Figure 1) [7,8]. Lymph formation and propulsion depend upon two different mechanisms, named “intrinsic and extrinsic.” They both affect local pressure gradients between interstitial spaces and vessel lumen and between adjacent lymphangions. The intrinsic mechanism, predominant in soft tissues such as the mesentery, relies on the lymphatic muscle (LM) net [9,10,11,12] surrounding the collecting vessel wall. Lymphatic muscle cells spontaneously contract by means of electrical commands arising in pacemaker cells within the lymphatic muscle layer, due to spontaneous transient depolarizations (STDs, induced by calcium-dependent chloride currents) or I_f_-like currents mediated by HCN channels such as it occurs in the heart [13,14,15,16,17]. The electrical signal then propagates to adjacent LM cells along the lymphatic network through GAP junctions [18]. Conversely, the extrinsic mechanism depends upon mechanical stresses originating in surrounding tissues [19] and transmitted to the vessel wall through fibrous extracellular matrix components [20,21,22]. This mechanism is predominant in lymphatics located in tissues undergoing significant cyclic movements, as the heart or skeletal muscular tissue [21], or is due to cardiogenic or respiratory-induced displacement [23,24]. Arteriolar vasomotion [25] and external compression [26] also affect lymphatic function, eventually inducing rhythmic vessel compression and expansion (Figure 1).

Extrinsic and intrinsic mechanisms may coexist in several areas of the body, and their relative importance is variable since the sources of extrinsic pumping may vary in strength from blood vessel vasomotion caused by the pulsatile blood flow to external forces coming from nearby muscles, which contract and relax, to relatively small contributions in the mesentery. Thoracic cavity is one region of the body where the continuous rhythmic activity of the heart and respiration gives rise to a constant interplay of the two lymphatic pumping mechanisms. In the rat diaphragm, both extrinsic and intrinsic mechanisms cooperate [27], as the extrinsic rhythmic respiratory movements mainly sustain lymphatic propulsion in the central tendon and in the medial muscular region. In this regard, longitudinal rather than perpendicular lymphatic orientation [28] with respect to muscle fibers arrangement results in functional differences. On the contrary, LM intrinsic spontaneous contractions are typical of vessels located at the far muscle periphery [29], where the extrinsic pump is less efficient probably due to the anisotropic stress distribution in the diaphragmatic dome [30]. Moreover, the LM mesh surrounding diaphragmatic lymphatics is not uniformly distributed and/or properly organized along the entire lymphatic network [29,31,32], since not contracting and/or passively distending sites can be found next to contracting ones [29].

The intrinsic myogenic activity can be modulated by a complex interplay of different signals, coming from both the vessel lumen and the surrounding interstitium. Moreover, in recent years, literature has provided evidence of a further neuronal modulation exerted by the autonomous nervous system on the intrinsic contractility of lymphatic vessels [33,34], which can introduce a further level of modulation of the response that lymphatic vessels, per se, can provide to stimuli coming from the tissue surrounding them. In this review, we focus our attention on the known physical stimuli and their putative sensing mechanisms localized on the lymphatic vessel wall that can affect spontaneous contractility.

## 2. Hydraulic Pressure Changes

The lymphatic system drains and propels lymph, mainly acting against a peripheral to central hydraulic pressure gradient. Thus, the competent intraluminal valve system ensures unidirectional fluid flow along the lymphatic network, minimizing lymph backflow [35,36,37,38]. Intrinsically and/or extrinsically induced oscillatory intraluminal pressure gradients (Δ*P_Lymph_*) force valve leaflets into an open/closed cycle to guarantee lymph propulsion in a step by step way. However, in some cases, intraluminal valves can be noncompetent, being biased in a partial open position, allowing some lymph backflow and fluid recirculation [5]. In lymphatic vessels of the diaphragm, this behavior can be clearly seen in peripheral lymphatic loops [27]. In the absence of a central pump, the lymphatic system thus relies on intrinsic and extrinsic mechanisms to sustain time-variable local lymphatic pressure gradients. Indeed, phasic contractions compress the lumen, driving fluid movement, and the unidirectional valve system yields net antegrade flow in most body areas, such as the mesentery.

Extrinsic contractions give rise to the highest pressure gradients between consecutive lymphangions, which result in a major effect on lymph flow. In fact, experiments performed on sheep hindlimb [26] showed that external forces acting on tissues surrounding lymphatic vessels may account for a fourfold increase in lymph flow with respect to lymphatic intrinsic contractility alone. Similarly, in the rat diaphragm the skeletal muscle contractions can induce an increase in up to 10-fold of lymph flow [27]. In collecting diaphragmatic lymphatics, end-expiratory intraluminal pressure is ~4 cm H_2_O; inspiratory diaphragmatic contraction significantly lowers intraluminal pressure in submesothelial vessels and in deeper parallel-oriented ones (down to −20 cm H_2_O), thus increasing the transmural pressure gradient (Δ*P_TM_*) and enhancing drainage of both interstitial and pleural fluids. In contrast, in deep transverse-oriented vessels, inspiratory contractions increase intraluminal pressure (~+10 cm H_2_O), facilitating lymph propulsion towards adjacent lymphangions [28].

On the other hand, intrinsic contractility plays a pivotal role in maintaining lymphatic drainage in many body areas devoid of significant tissue displacement. Similar to the cardiac cycle, lymphatic spontaneous activity generates a systolic phase, with LM active contraction causing lymph propulsion between adjacent lymphangions, and a diastolic one, upon LM relaxation, favoring the refilling of lymphangion. This phasic activity can be quantified in terms of contraction frequency (CF) and ejection fraction or stroke volume (SV) [39,40]. Studies by Muthuchamy et al. [10] in rat lymphatic vessels of mesentery and the thoracic duct demonstrated that LM possesses a unique contractile machinery. Proteins typical of vascular smooth muscle (such as SMB smooth muscle myosin heavy chain) together with non-smooth elements such as the fetal cardiac/skeletal slow-twitch muscle-specific β-MHC (beta myosin heavy chain) confer to LM a fast contractile behavior. In absence of a characterization of the peculiar contractile and regulatory elements of LM machinery, it had been widely thought that LM cells resembled the vascular smooth muscle cells. However, as LM contains skeletal, cardiac, and smooth muscle contractile elements, it can no longer be regarded as “lymphatic smooth muscle,” but rather as “lymphatic muscle.” Spontaneous contractions sprout from pacemaker sites and then propagate along the lymphangion chain in a coordinated fashion [18]. This behavior squeezes lymph, which fills the following segment. Indeed, in order to propel lymph forward [12], each lymphangion only needs to overcome the outflow transvalve pressure imposed by the proximal one. During the systolic phase of an intrinsic contraction, the intraluminal hydraulic pressure increases, even in a restricted area [40], thus generating an intraluminal pressure gradient favoring lymph propulsion. In the mesentery, intrinsic contractions arise in the smaller vessels and then propagate to the larger collectors, whose baseline intraluminal pressure progressively becomes significantly higher. Intrinsic contractions in more distal vessels generate pulsatile pressure oscillations of about 2–4 cm H_2_O, whereas in more proximal vessels, pressure increases up to about 10–20 cm H_2_O [41]. In order to guarantee lymph propulsion against the hydraulic pressure gradient, lymphangions cooperate as a long in-series chain, since they would not be able to propel lymph for long distances if they worked as single pumps [18]. Intraluminal valves also contribute to the net antegrade lymph propulsion, as they interrupt the lymph fluid column and minimize lymph backflow [35]. In vivo experiments performed on rat mesenteric lymphatics show that a transvalve pressure gradient of about 1–1.5 cm H_2_O is enough to open valves, whereas they can withstand pressures up to about 27 cm H_2_O when trying to impose a retrograde flow [41].

Lymphatic vessels sense and actively respond to changes in transmural and intraluminal hydraulic pressure gradients. Transmural pressure gradients (intraluminal—interstitial pressure difference) acting across the lymphatic vessels wall deeply affect spontaneous contractility, inducing a self-adjustment of the pump function and directly regulating CF (i.e., chronotropic effect) and contraction amplitude (i.e., inotropic effect) to handle the fluid load. In fact, rat mesenteric lymphatics and thoracic duct display optimal pumping activity at a transmural pressure of 3–5 cm H_2_O [42,43]. The contraction rate and strength rise with increasing transmural pressure, having the maximal activity at 5 cm H_2_O [44], then lymphatic pump efficiency decreases [45]. In fact, an increase in transmural pressure above 6–9 cm H_2_O causes an overall reduction in lymph flow, attained with a further increase in CF but a decrease in SV. This negative inotropic modulation is not properly compensated by the positive chronotropic effect [44], thus reducing lymphatic pumping. This can be explained by the vessel wall overdistension, which limits the contraction strength, according to the bell-shaped length-tension relationship of LM [46,47]. Although regional differences can be found in the optimal transmural pressure at which lymphatics display the maximal activity, vessels belonging to different body regions behave similarly to mesenteric vessels in pressure-limited modulation of contractility [43,48]. An age-dependent reduction in lymphatic response to a wall-tension (hoop stress) increase has also been reported. Indeed, both negative inotropic and chronotropic responses were shown in thoracic ducts of older rats, causing a reduced lymphatic functionality [49].

## 3. Lymph Flow and Shear Stress

Local lymphatic flux adapts to cope with the tissue drainage requirements. For example, vascular fluid load and hemodilution determine, according to Starling’s Law [1], an increase in transvascular fluid fluxes and local tissue pressure, which, in turn, lead to enhanced lymphatic contractility. Thus, lymphatic functionality increases to remove excess interstitial fluid load and prevent edemagenic conditions. In this regard, plasma dilution induces both inotropic and chronotropic positive effects in lymphatic collectors [22,40], up to the attainment of the maximal drainage capacity and lymph flow rate, above which lymphedema may eventually develop.

Intrinsic and extrinsic mechanisms cooperate to guarantee an adequate lymph flow rate. However, any extrinsically induced modulation able to sustain the required lymph propulsion tends to reduce the intrinsic spontaneous lymphatic contractions. Indeed, lymphatic vessels display a flow-induced inhibition of the intrinsic pumping mechanism when a proper lymph propulsion is induced by extrinsic forces, by imposing increasing intraluminal pressure and lymph flow. An interesting study performed by Gashev et al. [43] clearly shows the effect of flow on lymphatic spontaneous activity in different areas of the body, affecting both CF and amplitude (i.e., diastolic–systolic diameter difference). Imposed low lymph flow rate, at 1 cm H_2_O pressure gradient, reduces lymphatic contractility of about 30–40% in mesenteric, femoral, and cervical lymphatics and in the thoracic duct. Moreover, when a higher lymph flow rate is imposed with a 5 cm H_2_O pressure gradient CF is significantly reduced of about 60% in mesenteric and femoral lymphatics, whereas it faces a 92% and a 98% reduction in cervical lymphatics and in the thoracic duct, respectively. The authors also describe a flow-induced reduction in contraction amplitude distributed in a regional manner, as summarized in Table 1. The most susceptible lymphatic vessel seems to be the thoracic duct (−8 at 1 cm H_2_O pressure gradient and −95% at 5 cm H_2_O), whereas in mesenteric lymphatics contraction amplitude decreases of about 17% and 64% at 1 and 5 cm H_2_O, respectively. Considering that intrinsic contractions of the LM are required to increase intraluminal transvalve pressure gradient (*P_L_*_,1_–*P_L_*_,2_, Figure 1) leading to valves opening, if *P_L_*_,1_ is sufficiently higher than *P_L_*_,2_, no further intrinsic contractions are necessary for lymph propulsion and spontaneous activity stops. Therefore, lymphatic vessels behave like conduits [50], thus making them more energy-efficient. Moreover, when the mesenteric vessel intrinsic contractility is completely abolished, the lymphatic tone is reduced and the diastolic lumen diameter significantly increases up to +23%, thus reducing lymphatic vessels resistance to lymph flow by about 56% [42].

Lymphatics belonging to different areas of the body display local anatomical and functional adaptations. For example, diaphragmatic lymphatics located at the skeletal muscle periphery are often organized in complex and interconnected loop-like structures [29,51] including spatially segregated lymphatic vessel tracts displaying intrinsic contractions. A few stretch-activated lymphatic sites are also present and actively contract [29], aiding lymph propulsion when incontinent intraluminal valves allow lymph backflow [27]. Those vessels do not seem to possess the spontaneous activity induced by the lymphatic pacemaker, nevertheless they can contract when lymph goes through, thus favoring the overall lymph propulsion.

In blood vascular vessels, flow-induced shear stress stimulates endothelial cells (ECs) to produce and release nitric oxide (NO), affecting smooth muscle cells functionality. Lymphatic ECs lining the inner lumen sense and respond to different stimuli acting on the vessel wall, such as the wall tension induced by transmural and/or intraluminal pressure and the inner wall shear stress. Such physical forces that EC membranes experience in vivo, greatly influence endothelial function and result in the activation of signaling pathways and physiological responses [52,53]. A well-characterized mechanism of shear stress sensing is through the lymphatic EC surface mechanosensory receptors at cell–cell junctions, such as adherens junctions, leading to the VEGFR3 phosphorylation and activation of intracellular signaling cascade [54]. In addition, lymphatic endothelial nitric oxide synthase (eNOS) produces NO, which plays a key role in the signaling cascade of lymph flow sensing [42,55,56]. Indeed, in lymphatic vessels, endothelial-released NO induces LM hyperpolarization and the reduction in membrane resistance by the activation of ATP-sensitive K^+^ channels via the cGMP intracellular pathway [57]. Moreover, NO independently also inhibits the lymphatic pacemaker primarily via cGMP production leading to the activation of both PKG and PKA [58]. Furthermore, the use of cGMP/PKG inhibitors reduced the flow-related sensitivity to shear stress and the extrinsic flow-related vessel relaxation of the thoracic duct [59].

An additional recently proposed mechanosensing mechanism involves members of the Piezo family, named Piezo1 and Piezo2, originally identified as mechanically activated ion channels [60]. Particularly, Piezo1 is known as a cell stretch and a fluid shear sensor, also critically involved in the development of the lymphatic system [61,62]. In fact, Piezo1-KO mice pups display a reduced density of mesenteric lymphatic vessels and lack mature valves at the branching points. Mice lacking Piezo1 also display pleural effusion, possibly leading to an impairment of respiratory function, which might lead to lethality. In addition, the loss-of-function mutations of human Piezo1 is involved in familial lymphatic dysfunctions [63,64]. Furthermore, LECs exhibit changes in cell morphology towards a more cuboidal shape when exposed in vitro to fluid shear stress. They also display shear stress-induced up-regulation of genes involved in lymphatic valve development, which is largely abrogated in Piezo1-KO mice [62]. Additional evidence shows that Piezo1 overexpression in HEK 293 cells induces shear stress-evoked Ca^2+^ entry in these shear stress-resistant cells [61,65].

**Figure 2:**
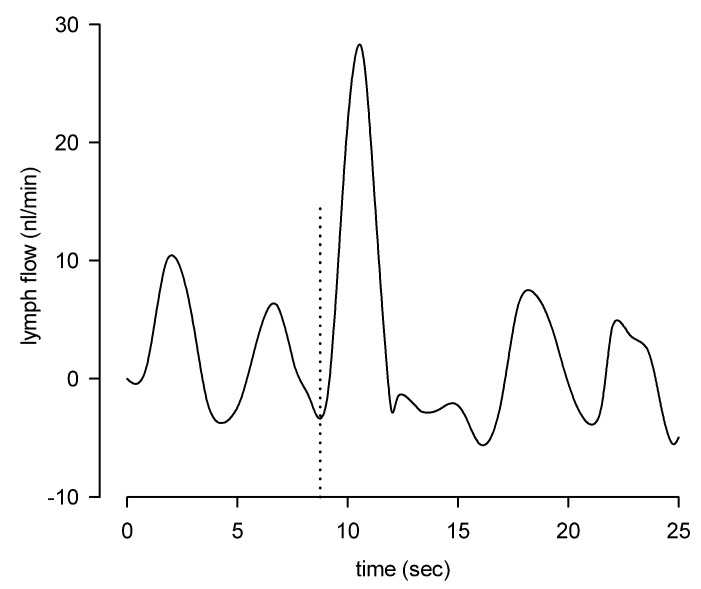
Modulation of lymphatic function: difference in oscillatory lymph flow induced by intrinsic contractions and one extrinsic contraction (experimentally induced at the time point indicated by the vertical dotted line).

Shear stress value can determine different responses in lymphatic vessels located in different areas of the body. Dixon and colleagues [38] evaluated in vivo the contraction to lymph velocity relationship in mesenteric lymphatics by tracking particles moving along the vessel. The wall shear stress was determined to be about 0.64 dyne/cm^2^, showing peaks of 4–12 dyne/cm^2^, approximately one-tenth lower than the one experienced by blood vessels, where it is lowest in large veins (less than 1 dyne/cm^2^) and increases up to 60–80 dyne/cm^2^ in small arterioles, with a remarkable example of the carotid bifurcation where, in the same vessel, depending on the site wall shear stress can vary between 1 and more than 600 dyne/cm^2^ [66]. Mukherjee et al. studied the wall shear stress dynamics in the rat thoracic duct in conditions of imposed flow at a transmural pressure of 3 cm H_2_O and with increasing intraluminal pressure up to 9 cm H_2_O. The wall shear stress was found to correlate with a decrease in both lymphatic tone (shown by the increased diastolic diameter) and CF. Such shear-related sensitivity highlights a “critical shear stress” (ranging between 0.1 and 10 dyne/cm^2^), representing the value at which lymphatic spontaneous CF decreases of about 50% [67]. In a different lymphatic district such as the rat diaphragm, we tracked the pathways of fluorescent microspheres to measure lymph velocity and then compute lymph flow and wall shear stress. Lymph flow was determined to be laminar both during intrinsic and extrinsic propulsion, as indicated by the very low Reynold’s number and the parabolic lymph velocity profile [27]. Moreover, in diaphragmatic vessels, flow-induced shear stress during intrinsic contractions was about 0.02 dyne/cm^2^, almost two orders of magnitude lower than in mesenteric or dermal lymphatics [68] and raised to about 0.25 dyne/cm^2^ during extrinsic contractions [27]. The very low shear stress experienced by the lymphatic endothelium may be the reason why no extrinsic-induced inhibition of intrinsic contractility has been found in diaphragmatic lymphatics (Figure 2). Indeed, even the higher shear stress induced by extrinsic contractions might be lower than the threshold for the induction of NO production [69,70,71].

## 4. Fluid Osmolarity in the Microenvironment

Changes in osmolarity of the interstitial fluid surrounding lymphatic vessels dramatically affect lymphatic intrinsic contractility and, therefore, lymph flow [72]. In rat diaphragmatic lymphatic vessels, acute exposition to changes in surrounding fluid osmolarity deeply affects the CF, without any inotropic effect, even in the typical range of plasma osmolarity, which in rats is about 288–336 mOsm [73]. This suggest that fluid osmolarity may critically modulate the lymphatic pacemaker activity without any significant effect on the LM machinery.

Hyposmolarity induces a two-phase response, in which an early (within ~5 min from the osmolarity decrease) rise in CF can be observed, followed by a later (up to ~14 min) decrease to an almost steady level of about 75% of the frequency in isosmotic conditions. Interestingly, the amplitude of the early CF peak increases in an osmolarity-dependent manner (+17.5% at 299 mOsm and +33.7% at 290 mOsm). Conversely, in hyperosmotic conditions, CF monotonically decreases, in an osmolarity-dependent manner, reaching a steady value of 46.3% at 315 mOsm and 29.5% at 324 mOsm [72], thus, suggesting the involvement of different mechanisms in the response to fluid hypo- or hyperosmolarity.

Focusing on the intriguing response to hyposmolarity, the early increase in lymphatic spontaneous contraction frequency and flow rate may reflect the need to rapidly remove the excess interstitial fluid, even in face of an unchanged contraction amplitude. This response is in line with the observation that several EC types and vascular smooth muscle cells express molecular mechanisms, which sense and respond to fluid osmolarity. Among them, the volume-regulated anion channels (VRACs) [74], and particularly ClC-3, are known to increase their opening probability in response to osmotic cell swelling [75,76] and may be involved in the response to the osmotic stress. Since in smooth muscle cells, the high chloride ions intracellular concentration exerts a depolarizing effect [77,78], the early increase in CF in an hyposmotic microenvironment may be related to the opening of VRACs expressed on lymphatic ECs and/or LM cell membranes, which, in turn, could eventually depolarize the LM, either directly or in an endothelium-dependent manner, thus increasing the speed of threshold crossing at the pacemaker sites. Moreover, lymphatic ECs possess transient receptor potential channels of the vanilloid family (TRPVs) [79,80]. Among them TRPV4 channels, which are widely expressed in the vasculature, can respond to a broad range of stimuli such as osmotic cell swelling, heat, and several ligands [81,82] by means of different mechanisms [83]. In ECs, the activation of TRPV4 by a selective agonist induces an increased concentration of calcium ions, which in turn activates IK_Ca_ and SK_Ca_ channels leading to cell hyperpolarization. The vessels’ functionality is thus regulated by smooth muscle cells hyperpolarization through myoendothelial junctions. The pivotal role played by vascular endothelial TRPV4 in modulation of smooth muscle activity is suggested by the nonselective TRPV channels blocker Ruthenium Red and the specific TRPV4 blocker HC-067047, which abolish the vascular response, such as it occurs when the endothelium is selectively ablated from the vessel wall [84]. Nevertheless, electrophysiological evidence have clarified that TRPV4 channels do not sense hyposmolarity per se, but rather the membrane stretching due to cell swelling. In fact, these channels are not activated when a hypotonic solution is simultaneously administered both on the intracellular and the extracellular side of the membrane in excised-patch experiments [85], where cell swelling does not occur. Hence, TRPV4 channels might play a crucial role in response to hyposmolarity by serving as volume-sensing receptors rather than proper osmoreceptors.

Concerning the response to hyperosmolarity and the related cell-shrinkage, it is well known that an increase in fluid osmolarity can lead to vascular EC and smooth muscle cells hyperpolarization, causing vasodilation [86] and lymph flow reduction in perfused cat mesenteric vessels [87]. Several mechanisms have been proposed, involving aquaporins, K_ATP_ ion channels, sodium pump, or VRACs inhibition [75,88,89,90,91]. The great variability in mechanisms, which can play the critical role, may be due to a very cell-specific interaction among VRACs, potassium channels, and other signaling mechanisms activated in the presence of a hyperosmotic environment.

Hyperosmotic stresses do not likely depend upon changes in plasma or tissue proteins but, rather, upon formation of molecular debris, normally absent in the tissue, released by fragmentation of larger tissue molecules. For example, development of even mild hydraulic or lesional edema is accompanied by significant fragmentation of proteoglycans of the extracellular matrix [47,92,93], leading to an increase in smaller molecular debris concentrations proportional to tissue damage. The coexistence of a factor upregulating lymph flow (increased interstitial pressure) with the hyperosmotic-dependent CF reduction might be an example of the interaction between independent mechanism operating at tissue level to optimize lymphatic CF and SV to face the tissue perturbation.

## 5. Local Tissue Temperature

**Figure 3:**
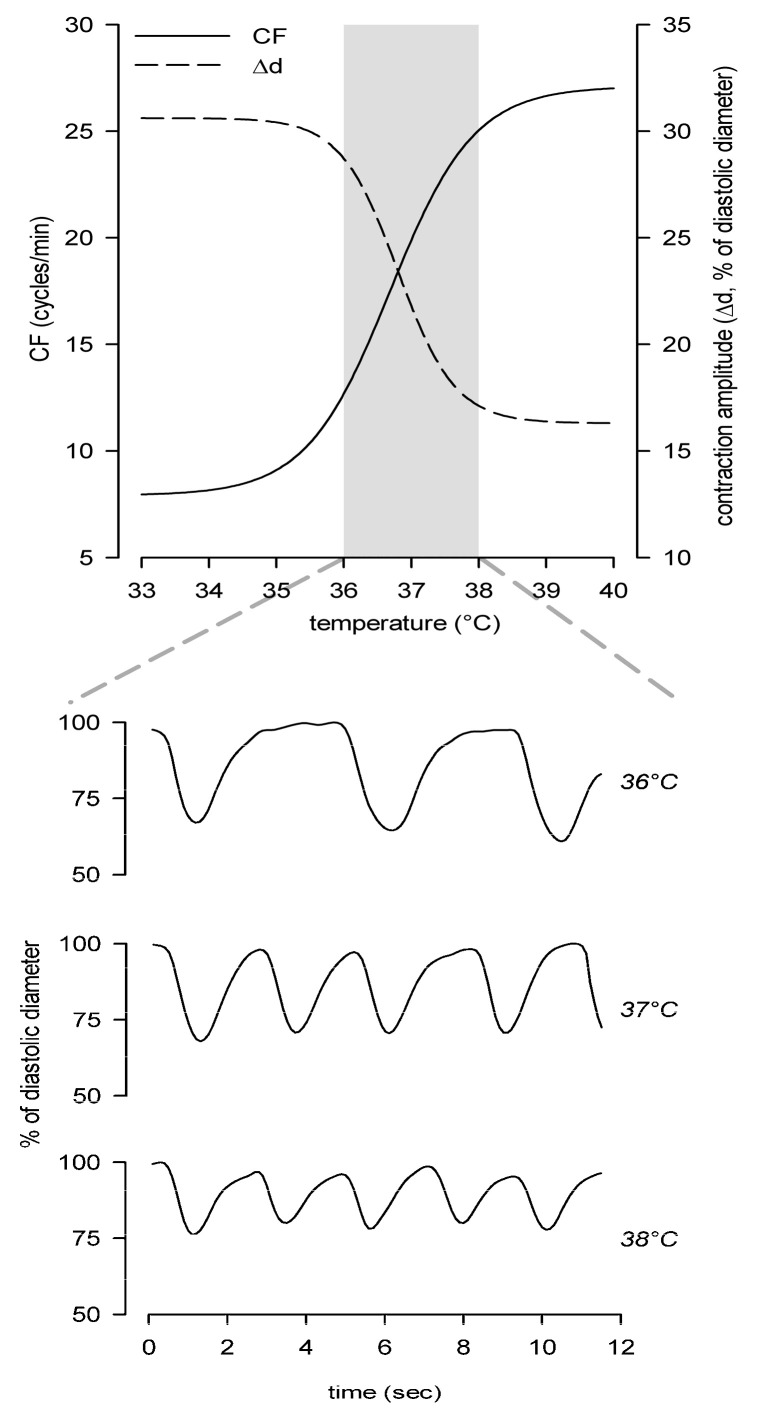
Temperature-dependent modulation of diaphragmatic lymphatics contractility, affecting both CF and contraction amplitude (Δd). Upper panel: positive chronotropic effect on *CF* (solid line) and negative inotropic effect on Δd (dashed line). Gray area highlights the range of steepest variation of both parameters. Lower panel shows representative traces of vessel diameter over time recorded in the range of 36–38 °C. Data taken from [94].

Even slight changes in temperature around the optimal set point deeply affect intrinsic contraction frequency and amplitude of lymphatic vessels belonging to different body districts, inside or outside the body thermal core, thus altering lymph formation and propulsion. In diaphragmatic lymphatics lying in the body thermal core at a constant temperature of ~37 °C, the increase in temperature in the range of 33–40 °C exerts at the same time a positive chronotropic effect and an opposite negative inotropic effect, with the net result of reducing SV (Figure 3). However, lymph flow increases with temperature, highlighting that intrinsic CF and not SV is the main factor affecting temperature-related fluid flow modulation. Similar results were obtained in lymphatics of the skin, where temperature increase in the range of 24–40 °C deeply affects intrinsic CF (i.e., positive chronotropic effect) without any inotropic effect [94]. The same temperature modulation of contraction rate is registered in the range of 27–40 °C in peripheral mesenteric lymphatics, which display a positive chronotropic response [95].

In this context, the existence of a temperature-sensing mechanism in diaphragmatic lymphatics represents some kind of novelty, as vessels located in the thermal core are likely exposed to a constant temperature, opposed to vessels located outside the thermal core and thus exposed to a wider range of temperatures. Temperature-dependency of physiological mechanisms can be evaluated by the temperature coefficient *Q*_10_, which provides the reaction rate increase for every 10 °C rise in temperature [96]. The rate of intrinsic lymphatic *CF* change upon increasing temperatures can be computed as in Equation (1):

(1) $$Q_{10}={\left(\frac{CF_{T_{2}}}{CF_{T_{1}}}\right)}^{\left(\frac{10}{T_{2}-T_{1}}\right)}$$

where *CF_T_*_2_ and *CF_T_*_1_ are the contraction frequencies recorded at *T*_2_ and *T*_1_, respectively [80]. In diaphragmatic lymphatics, the very steep increase in *CF* with temperature results in *Q*_10_ values up to 30. This suggests the existence of a complex regulatory mechanism involving temperature-sensing proteins and/or receptors, such as TRPV channels, the temperature sensors in different tissues [97,98]. Diaphragmatic lymphatics possess at least TRPV1, TRPV3, and TRPV4 channels [80], whose temperature ranges of activation encompass the physiological temperature of the body thermal core and display high *Q*_10_ values [99]. Among them, TRPV4 channels have been recently identified as a temperature sensor in diaphragmatic lymphatics. Ruthenium Red and HC-067047 abolish the chronotropic effect exerted by increasing the temperature, whereas the specific TRPV4 agonist GSK1016790A mirrors the positive chronotropic and negative inotropic effects induced by increasing temperatures.

In contrast, dermal and mesenteric lymphatics display the common heat response of most enzymatic processes and the temperature effect on ion channel gating [100]. Those vessels display low Q_10_ values, in the range of 1–3 and about 4 [94,95], respectively. Such *Q*_10_ values are compatible with the temperature effect on HCN channels of guinea pig ileal and rabbit cardiac muscle [101,102], critically involved in lymphatic intrinsic pacemaking [17]. This suggests no TRPVs involvement in the response to temperature of dermal and mesenteric lymphatics. However, an EC-LM interplay is hypothesized as the putative temperature-sensing mechanism in mesenteric lymphatics. In fact, the EC firing frequency suggests a clear temperature-dependency and the electrical activity directly controls the rate of lymphatic contractions [95].

## 6. Conclusions

There is an increasing number of experimental evidence that shows how lymph transport is not a mere passive and automatic process, but indeed, it represents a highly sophisticated system of very specialized endothelial and muscle cells, which not only can coordinate an active contraction strategy along vessels to obtain lymph drainage and propulsion but also can proactively respond to local physical stimuli in order to optimize lymph transport segmentally and in real time. Although an increasing number of papers have started to investigate these phenomena at a cellular and molecular level, this field is lagging behind the great amount of data and numerical modelling regarding physiological description of macroscopic parameters associated with lymph drainage and propulsion.

Future research could take advantage of the recent discoveries on the molecular mechanisms and stimuli that can increase the lymphatic intrinsic pumping and use them to develop new drugs or aimed physical stimuli that could increase in the long term, the lymph drainage from areas of the body subjected to lymphedema.

## Figures and Tables

**Table 1:** Effect of imposed flow on intrinsic contractile activity of lymphatic vessels from different body areas of the rat.

Vessels	Imposed Flow Gradient (Δ cm H_2_O)	CF (Cycles min^−1^)	EF	FPF (min^−1^)
**Mesenteric lymphatics**	0	9.0 ± 1.6	0.59 ± 0.05	5.1 ± 0.7
	1	6.1 ± 18	0.49 ± 0.10	3.1 ± 0.7
	3	4.6 ± 2.1	0.27 ± 0.11	1.9 ± 0.9
	5	3.4 ± 1.5	0.23 ± 0.10	1.4 ± 0.8
**Femoral lymphatics**	0	15.2 ± 2.6	0.17 ± 0.03	2.5 ± 0.6
	1	9.7 ± 3.0	0.11 ± 0.03	1.5 ± 0.7
	3	7.7 ± 3.0	0.08 ± 0.03	1.1 ± 0.6
	5	5.0 ± 2.4	0.09 ± 0.04	0.4 ± 0.2
**Cervical lymphatics**	0	18.9 ± 2.7	0.11 ± 0.02	2.1 ± 0.5
	1	11.4 ± 3.8	0.08 ± 0.03	1.4 ± 0.5
	3	7.4 ± 3.6	0.02 ± 0.02	0.2 ± 0.2
	5	1.5 ± 1.5	0.02 ± 0.02	0.2 ± 0.2
**Thoracic duct**	0	4.6 ± 0.6	0.31 ± 0.03	1.4 ± 0.2
	1	2.8 ± 0.7	0.16 ± 0.04	0.5 ± 0.1
	3	0.4 ± 0.3	0.03 ± 0.02	0.4 ± 0.0
	5	0.1 ± 0.1	0.02 ± 0.02	0.01 ± 0.01

Parameters defining intrinsic contractility are defined similarly to those in the heart. EF = (d_d_^2^ − d_s_^2^)/d_d_^2^, defines the volume change rather than the diameter (amplitude) change; FPF = CF × EF, defines the fractional volume pumped per minute, an index of the lymph pump output [39]. Data for regional changes in lymphatic contractility, under different imposed flow conditions, taken from Gashev et al. [43] d_d_, diastolic diameter; d_s_, systolic diameter; CF, contraction frequency; EF, ejection fraction, FPF, fractional pump flow.
